# Small Bowel Volvulus Complicated by Bowel Infarction

**DOI:** 10.5334/jbsr.2789

**Published:** 2022-04-28

**Authors:** Birgitt Janssens, Adelard De Backer, Filip Vanhoenacker

**Affiliations:** 1 AZ Sint Lucas Gent, BE; 2 UZ Antwerpen, UZ Gent, AZ Sint-Maarten, BE

**Keywords:** abdominal computed tomography, small bowel volvulus, small bowel infarction

## Abstract

**Teaching Point:** Torsion of a segment of the small bowel and its mesentery, together with closed loop obstruction and absent enhancement of paper-thin small bowel walls, is an alarm sign for small bowel volvulus complicated by acute bowel infarction.

## Case History

A 50-year-old woman presented at the emergency department with sudden onset of severe abdominal pain. She had a medical history of laparotomy for appendectomy and sterilization. Clinical examination showed diffuse abdominal tenderness with rebound and absence of bowel sounds.

**Figure 1:**
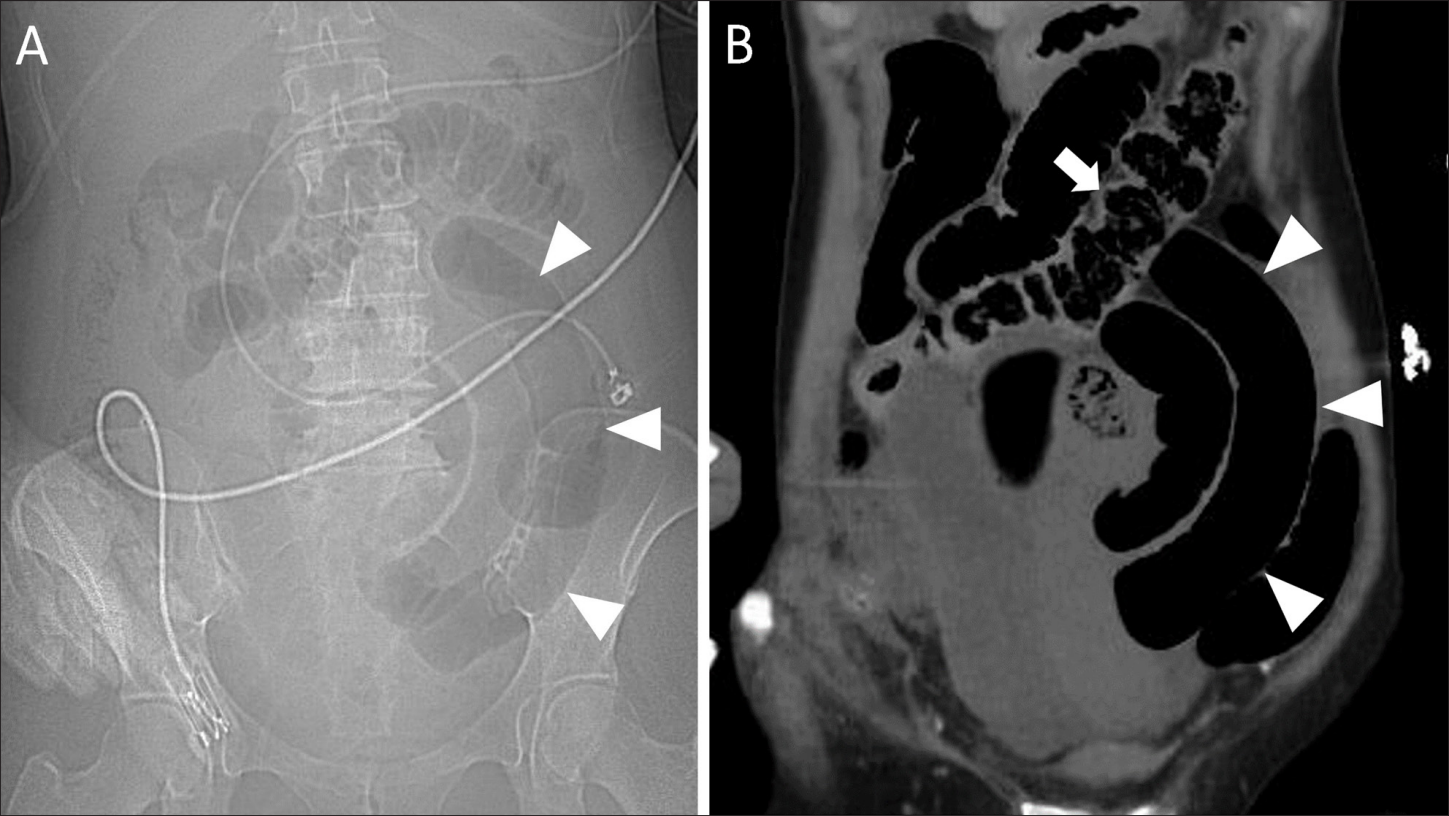


**Figure 2:**
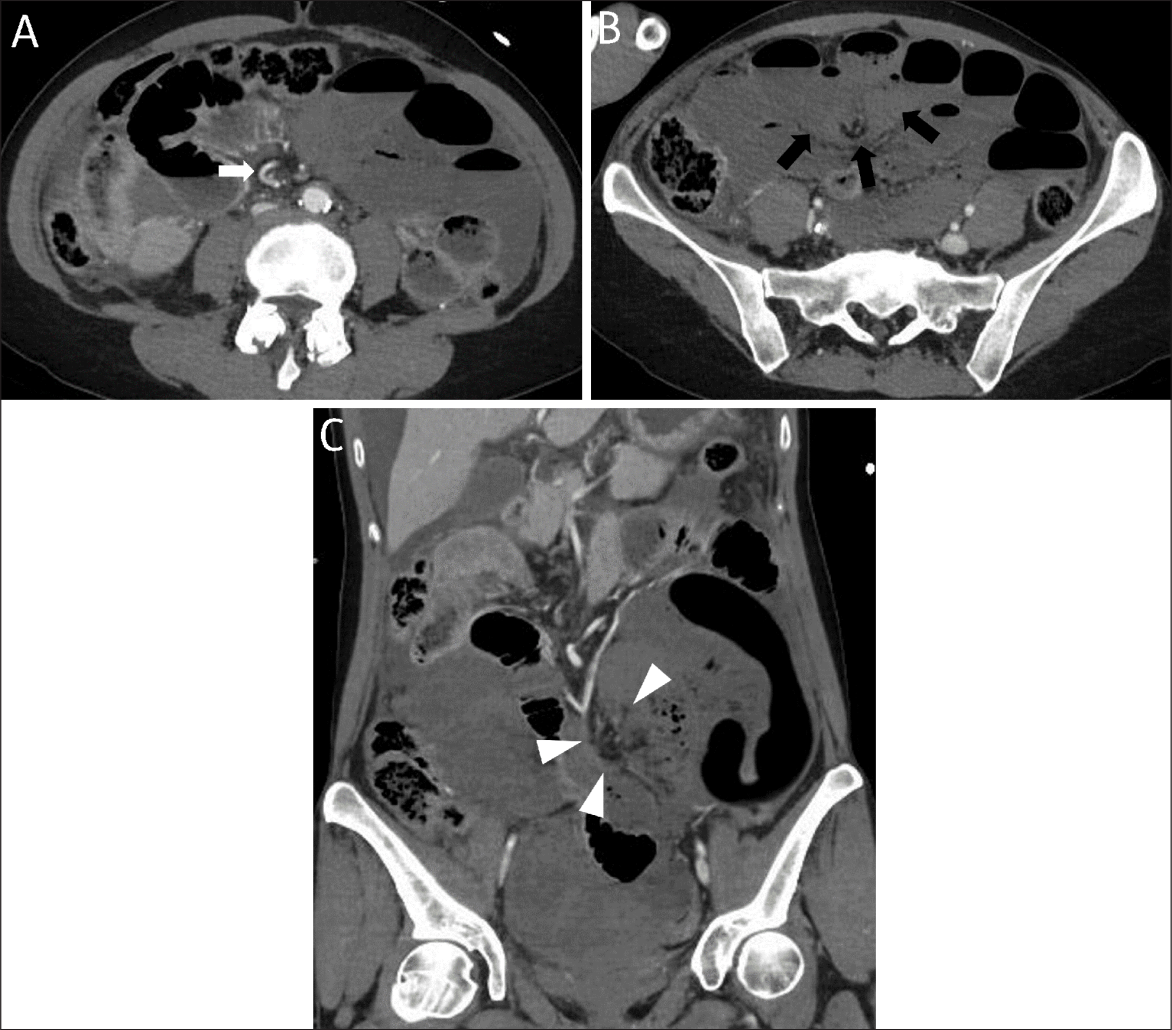


Scout view of abdominal computed tomography (CT) and coronal contrast-enhanced CT showed air-filled distended small bowel loops with a C-shaped configuration (***Figure 1A, B***, arrowheads) in the left hemi-abdomen and a nondistented colon (***Figure 1B***, white arrow). Axial images confirmed markedly small bowel dilatation with air-fluid levels consistent with small bowel obstruction. Twisting of the small bowel mesentery around the superior mesenteric artery resulted in a whirl sign (***Figure 2A***, white arrow). A double bird’s beak-sign converting to the compressed mesentery in the periumbilical region indicated a closed loop obstruction (***Figure 2B***, black arrows). Coronal images showed absence of contrast enhancement of paper-thin bowel walls, congested mesenteric vessels (***Figure 2C***, arrowheads) and hypertrophic collateral arteries indicating acute mesenteric infarction. There was intraperitoneal free fluid, but no signs of perforation. Laparatomy revealed a triple twisting of the mesenteric vasculature around its axis in presence of peritoneal adhesions, confirming a secondary small bowel volvulus (SBV).

## Comment

SBV complicated by acute infarction is an extremely rare but potentially life-threatening condition. Early recognition and prompt intervention are required to reduce mortality rate. In adults, SBV manifests mainly due to predisposing factors, known as secondary SBV.

SBV consists of an abnormal twisting of bowel loops around its own mesentery. Mesenteric torsion results in a closed loop obstruction and occlusion of the mesenteric vasculature with subsequent intestinal ischemia and finally necrosis. As the blood flow ceases to reach the small bowel, progressive ischemia with transmural infarction results in loss of muscle tone, which manifests as distended and thin-walled bowel loops [1].

CT is the imaging modality of choice to diagnose SBV complicated by acute infarction. Key findings include whirl sign, double bird’s beak sign, compression of the mesentery and its vessels, absence of contrast enhancement in paper-thin bowel walls, and closed loop obstruction. Non-enhancing paper-thin bowel walls is a specific sign of bowel ischemia with infarction. Other findings include air in the bowel wall (pneumatosis intestinalis), portal or mesenteric venous gas, mesenteric edema or engorgement of the mesenteric vessels, mesenteric haemorrhage, and intraperitoneal free fluid.

Treatment of SBV requires surgical intervention to reduce volvulus and re-establish blood flow. In case of ischemia with necrosis, small bowel resection is mandatory.
